# F222. CONCRETE THINKING PATTERN IN DAILY DECISION-MAKING PROCESS OF PATIENTS WITH SCHIZOPHRENIA: THROUGH EYE-TRACKING

**DOI:** 10.1093/schbul/sby017.753

**Published:** 2018-04-01

**Authors:** Soo-Jeong Kim, Yeon-Ju Hong, Min-Woo Kim, Young-Hoon Jung, Jae-Jin Kim

**Affiliations:** Yonsei University College of Medicine

## Abstract

**Background:**

Concrete thinking is one of the common clinical features of patients with schizophrenia, especially chronic and symptomatically severe patients. Until now, neuropsychological tests or proverb tests have been used to measure the difficulty of abstract thinking in patients with schizophrenia, but these methods have been difficult to know how concrete thinking patterns affect daily living of schizophrenia patients. In this study, we constructed a task that supposes the purchase of clothes in order to realize a situation close to the decision making of everyday life and tried to visualize the thinking process through eye-tracking method.

**Methods:**

Each twenty of healthy controls and schizophrenia patients performed the task of purchasing clothes. In serial, subjects are asked whether the clothes are fancy, suitable for themselves, affordable and whether they would buy the clothes. The eye gaze information was obtained by using the eye tracking system of the SensoMotoric Instrument, and the fixation time in the face and the clothes areas was measured. The changes of the fixation time ratio of the face to the clothes between the matching and the purchasing decision blocks for each group were compaired using repeated-measure ANOVA and the change of the ratio in patients with schizophrenia was analyzed with the PANSS concrete thinking score using generalized estimating equations. To investigate the relationship between the fixation time ratio and the answer of the purchasing block, two-way ANOVA with post hoc independent T-test was used.

**Results:**

There was a significant interaction effect between the groups on the change of the fixation time ratio(p<0.001). In the patient group, the fixation time ratio decreased (matching:0.474 ± 0.044, purchasing:0.206 ± 0.069) while the fixation time ratio increased in the control group (preference:0.233 ± 0.041, purchase decision:0.463 ± 0.064). To investigate whether the change of the fixation time ratio is affected by the degree of concrete thinking, the odds ratio of the concrete thinking score to the fixation time ratio was calculated as 0.825(95% CI; 0.684–0.992). The higher the concrete thinking score, the greater the decrease in the fixation time ratio was. Comparing the relationship between the answers in the purchasing block and the fixation time ratio, the interaction of fixation time ratios for each group was significant(p=0.031). In the post-hoc independent t-test, the fixation time ratio was significantly different for the control group(p<0.001); 0.167 ± 0.124 with purchase and 0.566 ± 0.073 without the purchase. For the patient group, there was no significant difference in the fixation time ratios by the answer.

**Discussion:**

The patients with schizophrenia relatively concentrated more on the face in the matching block, where the question included both “you” referring to the self and the clothes. In the purchasing block, where question did not contain “you”, they focused less on the face. The concrete thinking scores had a significant influence on the change of the fixation time ratio in the patients with schizophrenia, confirming the possibility that the concrete thinking contributed to the difference of gaze information patterns between the two groups. In the final purchasing block, the differences in fixation time ratios by the answer were observed only in the control group. If the price is higher than expected in the previous block, it is estimated that the negative emotion is triggered and schizophrenia patients could easily depend on concrete thinking rather than consolidating the information from the previous blocks and making a re-judgment. It is necessary for the further study to devise a task which can measure emotional reaction and also closely related with the decision of everyday life.

